# PW01-025 – Definition of colchicine resistance in FMF

**DOI:** 10.1186/1546-0096-11-S1-A78

**Published:** 2013-11-08

**Authors:** E Demirkaya, C Acikel, A Tufan, A Kucuk, A Berdeli, A Gul, AM Onat, A Delibas, A Duzova, A Dinc, O Yavascan, O Kasapcopur, B Makay, B Goker, B Sozeri, B Kisacik, E Comak, E Unsal, E Erken, E Gunal, E Baskin, F Yalcinkaya, F Yildiz, F Gok, G Basbozkurt, G Ozcelik, G Demircin, H Poyrazoglu, H Erdem, H Direskeneli, H Ozer, H Ozdogan, I Simsek, I Dursun, I Gokce, M Tunca, M Gurgoze, N Cakar, N Akinci, N Ayaz, O Donmez, O Ozkaya, R Topaloglu, S Kavukcu, S Yuksel, S Akar, S Bakkaloglu, S Emre, S Senel, S Erten, S Yavuz, S Kalman, T Kasifoglu, U Kalyoncu, Y Tabel, Z Ekinci, S Ozen

**Affiliations:** 1 Turkish FMF study group, FAVOR, Ankara, Turkey

## Introduction

Familial Mediterranean fever (FMF) is an autoinflammatory disorder, characterized by periodic fever and serosal inflammation. Colchicine is effective in controlling the attacks and preventing the development of amyloidosis. About 5-10% of the patients do not respond to colchicine.

## Objectives

In this study it was aimed to determine the set of criteria for the diagnosis of resistance to colchicine.

## Methods

This study was planned with Delphi technique sent to 70 experts on FMF and 59 of them approved to attend. In the first Delphi round, clinical and laboratory findings indicating colchicine resistance and the protocol which would define resistance to treatment and exclusion criteria were defined. Based on the results of the first Delphi, a second Delphi form which included 5 evaluation questions was developed. In this latter form the questions to be used in order to define complete response, partial response and non-response were tried to be determined.

## Results

In the first Delphi round, persistence of the frequency, severity and the duration of episodes in spite of the treatment with adequate dosage ranked the highest. Laboratory findings that are thought as the best indicator of resistance to treatment were high levels of acute phase reactants (In order of frequency; CRP, ESR, SAA and fibrinogen).

In the second Delphi round, among 57 experts, 35 experts reported the frequency of attacks while 10 experts reported the duration and 9 reported the severity of the disease as an indicator for the assessment of response to colchicine treatment.

For a complete response, normal CRP levels were required by 54 experts while 43 experts reported that 50% decrease in CRP levels could be accepted as partial response to treatment. Appropriate duration for the assessment of response to colchicine treatment was determined as 3 to 6 months by 34 experts. It was also stated that the highest dose for age and weight should be given in order to state colchicine resistance. Thirty three experts stated that the patient should be attack free for a complete response to colchicine treatment. For defining the partial response to treatment, a 50% decrease in attack frequency was the most favored choice.

## Conclusion

Assessing the colchicine resistance via concrete and agreed scale will provide a reliable data. The study is to be finalized soon with an expert panel.

## Disclosure of interest

None declared

